# Exploring the Development of Vaccine Platforms, With a Particular Focus on the COVID‐19 Pandemic

**DOI:** 10.1111/apa.70762

**Published:** 2026-09-24

**Authors:** Matti Sällberg

**Affiliations:** ^1^ Department of Laboratory Medicine Vice President KI Campus South Huddinge Sweden

**Keywords:** childhood deaths, genetic vaccines, immune response, limitations, pathogens

## Abstract

Vaccines have been a cornerstone of human health for more than 200 years and one of the most important tools to reduce childhood deaths caused by pathogens. The concept of vaccines is basically to take something safe, make it immunogenic and induce an immune response in the host that will protect it against something dangerous. Live attenuated vaccines were the only option two centuries ago. These are excellent, and possibly the best vaccine platform, but they have limitations as they cannot always be given to everyone safely, for example due to deficient immunity or pregnancy. That is why newer platforms, such as killed or inactivated vaccines, were developed. These provide better all‐round safety, but are often less immunogenic and require additional doses. Subcomponent vaccines were then developed in the 1980s. These only used parts of the virus, further improving safety and introducing the possibility of producing vaccines using gene technology. Adding genetic vaccines to the toolbox revolutionized the way that we handled the COVID‐19 pandemic. The host became the producer of the vaccine, which better mimicked the immune responses induced by the live attenuated vaccines. All these platforms will be discussed in this mini‐review.

## Key Points

- This mini‐review explores how vaccines have been a cornerstone of human health and one of the most important ways of reducing childhood deaths from pathogens for more than 200 years.
- It looks at how vaccines developed, from live vaccines to killed or inactivated vaccines, and addresses their limitations for certain patient groups.
- The review pays particular attention to how genetic vaccines revolutionized the way that the COVID‐19 pandemic was handled.
- The review highlights the misconceptions around vaccines and autism.

**Abbreviations**
AEs: adverse events
DNA: deoxyribonucleic acid
HBV: hepatitis B virus
MMR: measles mumps rubella
mRNA: messenger ribonucleic acid
RSV: respiratory syncytial virus
SARS‐CoV‐2: severe acute respiratory syndrome coronavirus 2

1

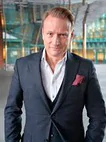

## 2 Introduction

Vaccines have reduced childhood deaths from pathogens for more than two centuries. But the COVID‐19 pandemic changed the face of vaccinology completely. Before the pandemic, vaccines against new viruses took 5–10 years to reach the market. They all used the old existing platforms, such as live attenuated, killed whole viruses, and subviral components. These were generally regarded as reliable and safe platforms and were the basis for human and veterinary vaccines. As vaccines are intended to prevent something that has not yet occurred in a naive individual, there is a much higher safety bar than treatments for an ongoing disease. That is why new vaccines or adjuvants have been slowly introduced into clinical practice.

Genetic vaccines became the new technology when COVID‐19 arrived. They had been lurking in the background in the vaccine field for 10–20 years and had been used in clinical trials up to phase II. The three first vaccines approved in the Europe and the USA were all genetic vaccines, based on messenger ribonucleic acid (mRNA), or adenovirus (Ad)‐based viral vectors. The vaccines were formulated in lipid nanoparticles or in adenoviral vectors from humans or chimpanzees and all coded for the spike protein of the severe acute respiratory syndrome coronavirus 2 (SARS‐CoV‐2). They all received conditional approval within 12 months of the genetic code of the virus being made public. This was an exceptional achievement that required extensive collaboration between academia, industry, the healthcare sector and regulatory authorities. This mini review discusses the different platforms that are currently used for human vaccines and what we may expect in the future.

## 3 Classic Vaccine Platforms

### 3.1 Live Attenuated Viral Vaccines

These are by far the oldest vaccine platforms, and the first one was developed for the smallpox vaccine. The earliest known form of smallpox vaccination was called variolation and it was first described in China and India. This basically meant that it induced a mild smallpox infection using a low amount of the virus [1]. However, this sometimes resulted in a full‐blown and deadly smallpox infection and this meant that safety was a major issue. In the late 1700s Edward Jenner used the cowpox virus to prevent smallpox [2]. He had observed that milkmaids seldom succumbed to smallpox and decided to find out why. He took liquid from the blistered hands of milkmaids who were infected by the cowpox virus and injected it into eight‐year‐old James Phipps, the son of one of his employees. When James had recovered from cowpox, Jenner exposed him to smallpox [2]. The boy survived and this completely changed the face of the smallpox epidemic. It also played a key role in its eradication in the late 1970s.

A live attenuated vaccine generally consists of a live virus that has been attenuated by serial passage in tissue culture (Figure 1). The attenuated and now non‐pathogenic virus infects cells and activates the immune system (Figure 1). A classic example was the oral polio vaccine, which was originally developed by Albert Sabin in the 1950s [3]. The immune responses triggered by the live attenuated vaccine then protected the person against the infection, or disease, caused by the pathogenic form of the virus. The advantages of live attenuated vaccines are that they are probably the most potent form of vaccines. This is because they trigger a strong response, consisting of B cells, CD4+ T cells and CD8+ T cells and a strong immunological memory [4]. A single vaccination can often result in lifelong immunity against the pathogen. The reason for this strong immune activation is that the live attenuated virus completes a full viral life cycle, which activates both the innate and the acquired immune response and triggers all the danger signals that an infection does [4].

**FIGURE 1:**
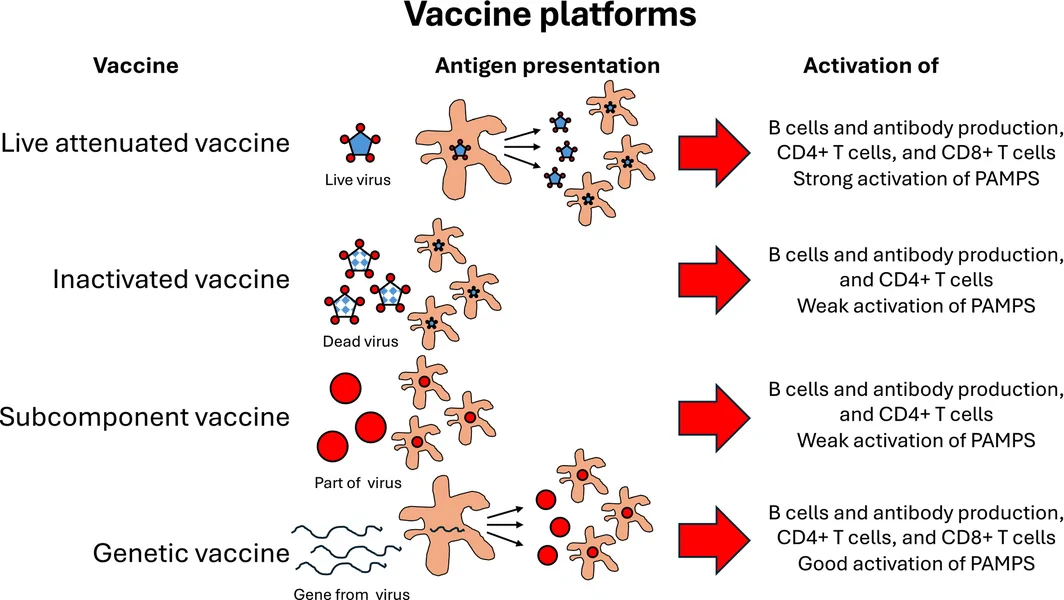
Cartoon illustrating the mode of immune activation by the four major vaccine platforms.

Examples of live attenuated vaccines that are used today are the measles, mumps, and rubella (MMR) triple vaccine, the oral polio vaccine, the yellow fever vaccine, one of the recent Chikungunya vaccines, and the chickenpox vaccine. Chickenpox vaccines were initially developed in Japan [5], but first implemented on a large scale in America in 1995 [6]. As these are childhood vaccines, they have also been shown to reduce herpes zoster, or shingles, in children under 18 [7]. It remains to be seen if the childhood vaccinations also prevent or result in milder cases of shingles in adults and older people. We know that vaccinating people when they are older can do this [8]. However, as these childhood vaccinations only started in 1996 in America, it will be another 20–40 years before we know for sure.

So what are the risks and why are we not using this platform more? A major issue is the risk of the attenuated virus causing a disease in a host with mild or severe immune dysfunction. For example, we know that the live oral polio vaccine can cause an estimated one case of polio per 1 million doses [9]. This means that it is beneficial in high endemic areas but not acceptable in low endemic areas. Thus, this is one of the major issues with the platform. Live vaccines are avoided during pregnancy, and maternal immunity may impair the immunogenicity in a young child. That is why the mumps, measles and rubella (MMR) vaccine is given mainly at 12–18 months of age. In addition, it may be too dangerous to generate attenuated forms of some viruses, as they may revert to a wild type or recombine with wild type viruses.

### 3.2 Killed Whole Virus Vaccines

The first killed or inactivated bacterial vaccines were developed in the late 19th century for cholera and the plague. The viruses are grown in various culture systems and inactivated in a number of ways, including heat, different forms of irradiation, formaldehyde or beta‐propiolactone. The inactivated vaccine is then administered to activate the immune response (Figure 1). The first killed viral vaccine, the injectable polio vaccine, was developed by Salk in the 1950s. This was also the decade when the first vaccine tradegy occurred. In 1955, Cutter Laboratories released a batch of polio vaccine that contained the live polio virus and that caused a number of polio cases and deaths in America [10]. It was the first case of a vaccine failure, and it is now erroneously used by the antivaccine movement to describe the dangers of today's vaccines. Importantly, the incident led to stronger regulations for the development and release of vaccines, which means that we now have safer vaccines. The Sabin and Salk vaccines have been successfully used to control and almost eradicate polio on a global scale. A second vaccine event was the formalin inactivated respiratory syncytial virus (RSV) vaccine that was evaluated in a small clinical trial in children in 1965. Today that would be regarded as a phase 1 trial [11]. Almost two‐thirds (65%) of the vaccine recipients became infected with the RSV and, of these, 80% were hospitalized. In contrast, only 5% of the controls who received a parainfluenza virus vaccine became infected with parainfluenza [11]. Thus, the RSV vaccine only worsened the symptoms. This became known as vaccine associated enhanced respiratory disease [12] and contributed to the development of the vaccine adverse event reporting system at the American Centers for Disease Control. Current RSV vaccines have addressed this problem, but they are still only approved for adults in risk groups, those above 60 years of age, and in pregnant mothers between weeks 24–36 to protect the child after birth through maternal antibodies to RSV. Clinical trials of RSV vaccines in children are still ongoing, but some have been halted due to safety concerns. The field is moving forward with caution.

Safety is a clear advantage of inactivated and killed vaccines, because the virus is dead. However, a disadvantage is weaker immunogenicity, as multiple doses of the vaccine are needed. Therefore, these types of vaccines are often mixed with an adjuvant, namely an immunostimulating compound. Examples of inactivated and killed vaccines are the Chinese COVID‐19 vaccine, influenza vaccine, hepatitis A vaccine, injectable polio vaccine, and varicella zoster vaccine.

### 3.3 Subcomponent Vaccines

A subcomponent vaccine only contains one or several parts of the virus. They can be formulated in different ways. The virus can be grown in cells and then the desired viral protein or proteins are purified and included in the vaccine. Alternatively, the gene encoding the viral protein is inserted into a bacterial, insect, or animal cell line from which the protein is expressed, purified, and used in the vaccine. The most commonly used components are the proteins involved in receptor binding, meaning that they can induce both neutralizing antibodies and T cells against the same proteins. The first subcomponent vaccine was the hepatitis B vaccine, which originally consisted of hepatitis B virus (HBV) surface antigen particles that were purified from people with chronic HBV infections. The HBV vaccine was the first approved vaccine produced by genetic modification of yeast cells, which allowed glycosylation of the antigen. This was done for safety reasons and better general acceptance than a vaccine purified from human plasma. The HBV has been an extremely effective childhood vaccine and we now know it reduces the risk of hepatocellular carcinomas. When the HBV vaccine is given at birth, it has a > 90% protective effect despite that the child is vertically infected by HBV from the mother at the same time. Thus, this is truly a highly effective vaccine.

Other subcomponent vaccines are the split influenza vaccine, the COVID‐19 vaccine from Novavax, and the human papillomavirus vaccine. The latter is an interesting subcomponent vaccine, as it contains only one protein that will assemble into virus‐like particles (VLPs). The advantage of vaccines based on such VLPs is that they are multimeric and effectively engage and activate both B and T cells. This means that the subcomponent platform is often highly immunogenic.

## 4 New Vaccine Platforms

### 4.1 Genetic Vaccines

Genetic vaccines have been explored in animal studies and early clinical trials for the past 30 years. Researchers showed that just injecting a deoxyribonucleic acid (DNA) or mRNA into a cell in vivo resulted in an immune response to the expressed protein. However, the major breakthrough was the genetic vaccine developed to tackle the SARS‐CoV‐2 infection during the COVID‐19 pandemic. The two major genetic vaccines were mRNA formulated in lipid nanoparticles that enabled cellular uptake and different types of adenoviral vectors. The latter is a non‐replicating virus that delivers the vaccine gene as a DNA to the nucleus of the cells and then transcribes it to mRNA and transports it to the cytoplasm for translation of the vaccine protein. The mRNA formulated in lipid nanoparticles (LNPs) is taken up and released in the cytoplasm and directly translated into the vaccine protein. A third modality that was evaluated and mainly used in India was DNA vaccines. Basically, a DNA plasmid containing the vaccine gene had to enter the cell and its nucleus had to be transcribed to mRNA and then translated into the vaccine protein.

The advantage of both mRNA‐ and DNA‐based vaccines is that they can be given to a patient an unlimited number of times, as neither induce antibodies to the delivery platform. In contrast, adenoviral and pox vectors contain the receptor binding part on their surface, and this induces antibodies that neutralize the vector after a single dose. Thus, there is a significant problem with delivering the multiple doses that are often needed with this type of vaccines, unlike live attenuated viruses that replicate and activate numerous immune signals.

## 5 Combining Different Vaccine Platforms

An obvious next step in vaccine development is to combine different vaccine platforms in so called heterologous prime‐boost strategies. Several animal studies have shown that combining vaccinations based on different platforms can induce broader and stronger immune responses than using a single platform. This is particularly true for viral vectors, such as adeno and poxvirus vectors. This is when the first vaccination induces immune responses to the viral vector used to deliver the vaccine that impairs the efficiency of a second dose. The advantage of both platforms can be used by combining these with mRNA or DNA vaccines, which can be given an infinite number of times. During the COVID‐19 pandemic, several studies showed that a first dose of a viral vectored vaccine was greatly improved by mRNA [13, 14]. We have also shown that three doses of mRNA vaccines followed by a DNA vaccine booster dose broadened immune responses during the COVID‐19 pandemic [15]. The mRNA vaccines encoded the spike protein and were followed by a DNA vaccine that encoded parts of the spike combined with the membrane and the nucleoprotein of SARS‐CoV‐2 [15].

## 6 Artificial Intelligence and Machine Learning

There is little doubt that machine learning, or artificial intelligence (AI), will play a crucial role in future vaccine development. These use large language models that have been adapted to genetic and genomic data. For example, it has been shown that training AI using millions of bacteriophage genomes could generate completely new viable bacteriophages. These could overcome bacterial resistance to bacteriophages more effectively than existing ones [16]. It is possible that more advanced pathogens, such as eukaryotic viruses, could soon be tailor‐made to create synthetic attenuated vaccines using AI. This may be the beginning of a new era in vaccinology.

## 7 Adverse Events

All vaccines induce reactogenicity and adverse events (AEs), such as local redness, swelling, and tenderness or pain. This is due to the immediate local inflammatory response. The more noticeable AEs are fatigue, flu‐like symptoms with fever, headaches, and muscle aches. These normally resolve within 24 h, but in some cases may last for some days. Again, this is due to the immune activation induced by the vaccine. In particular, live attenuated vaccines tend to cause pronounced but mild AEs because they activate multiple immune signals that represent the virus infection.

The COVID‐19 vaccines provide good illustrations of how AEs were recorded and discovered. The genetic vaccines used in the COVID‐19 pandemic were evaluated in large phase 3 clinical trials before they received conditional approval [17, 18, 19]. These trials showed that the vaccines caused AEs that were similar to existing vaccines and in particular the live attenuated vaccines. That was not surprising, since genetic vaccines are similar to live attenuated vaccines in that they enter the cell and activate multiple signal pathways. In addition, the presence of mRNA outside the cells at the injection site contributes to local inflammation. Thus, although these represent new vaccine technologies, the AEs were largely the same. However, rare AEs are not detected until millions of subjects have been vaccinated and rare AEs related to both adeno‐based and mRNA‐based COVID‐19 vaccines have been recorded. With regard to adeno‐based vector‐based vaccines, it was observed that 2–3 out of 100 000 women developed blood clots or thrombosis with thrombocytopenia syndrome in the brain [20]. That is why the vaccine was not used for younger people and it was later withdrawn in many countries. However, it is estimated that the adeno‐based vaccines saved millions of lives and supported their overall safety. With respect to the mRNA vaccines, the major rare and severe AE was transient myocarditis or pericarditis in around 5 per million young men [21]. As stated, it would not have been possible to clearly identify either of these more severe AEs in advance, even in large phase 3 clinical trials. This was similar to the swine flu vaccine used in 2009, which was implicated in rare cases of narcolepsy in the Nordic countries and the UK. This could have been caused by an autoimmune reaction because the vaccine contained a strong adjuvant [22]. However, it should be noted that reports from China and Taiwan also suggested associations between the influenza virus infection and narcolepsy [23, 24].

## 8 Vaccines do Not Cause Autism

A major misconception about vaccines was caused by a study performed by former UK Doctor Andrew Wakefield, which was published in the Lancet in 1998 [25] and retracted in 2010 [26]. Wakefield studied 12 children and stated that the parents of eight of those said that their children developed autism after they received the MMR vaccination [25]. In 2004, the Sunday Times published an article by investigative journalist Brian Deer. This article revealed that the Wakefield study lacked regulatory approvals and that the study findings had been misrepresented because the paper hid the fact that the subjects had been highly selected. In addition, Wakefield had received funding from a lawyer actively suing pharmaceutical companies on the notion that the MMR vaccine caused autism. Neither the journal nor his co‐authors were infomed about this fact. Deer later wrote a series of detailed articles in the British Medical Journal in 2010–2011, which documented what had happened [27, 28, 29, 30, 31, 32]. Already within the first year from Wakefield's publication in 1998, studies were published that refuted Wakefields story [33], and these were strongly supported by larger studies [34, 35]. In 2004, 10 of the 13 authors of the original paper retracted the claim that the MMR vaccine caused autism [36]. Thus, taken together, there is no scientific support for that the MMR vaccine causes autism. It took unfortunately until 2010 until the journal finally retracted the paper. During these years this paper created a completely unfounded vaccine scare in the UK that spread to other parts of the world. In 2010 the UK General Medical Council erased Wakefield's name from the medical register. This is a really unfortunate example of how a scientifically flawed publication, which has been clearly and convincingly refuted, still has repercussions almost 30 years later. That is truly sad. As a final comment, it is to be expected that some children given the MMR vaccine will be diagnosed with autism soon after the vaccination, simply because these events coincide in time, but this does by no means imply that there is a causal relationship between these two events.

## 9 Conclusion

The development of new vaccine platforms provides vital protection against viral infections. As the COVID‐19 pandemic demonstrated, it is possible to develop new, highly effective vaccine technologies that can be rapidly applied to large‐scale vaccine trials and globally distributed. What was unique about the COVID‐19 pandemic was that it was the second severe acute respiratory syndrome to be identified. The first, in 2003, had only caused a small rapidly contained outbreak that was not classed as a pandemic. But SARS‐CoV‐2 was more infectious and rapidly gained pandemic status. Previous pandemics had been caused by influenza viruses, such as the 2009 swine flu pandemic, and the technology required to make rapid vaccines for influenza already existed. Being able to respond rapidly is extremely important when new viruses, or other pathogens, cause pandemics. That is why it is vital that we have new platforms that can rapidly generate new viable vaccine candidates that are safe, effective and suitable for large‐scale production. This will provide the cornerstone we need to save lives during future outbreaks and pandemics.

## Author Contributions

**Matti Sällberg:** conceptualization, writing – original draft, writing – review and editing, visualization, funding acquisition, project administration.

## Funding

Research supported by: Cancerfonden * 23‐3071 Vetenskapsrådet * 2025‐06729.

## Conflicts of Interest

Matti Sällberg is a co‐founder and board member of SVF Vaccines AB, Sweden and gives lectures and/or consults for different companies with interests in vaccines, including Intervacc, Pfizer, GSK, and Moderna.

## Data Availability

The data that support the findings of this study are available on request from the corresponding author. The data are not publicly available due to privacy or ethical restrictions.
